# Natural abundance isotope ratios to differentiate sources of carbon used during tumor growth in vivo

**DOI:** 10.1186/s12915-021-01012-5

**Published:** 2021-05-10

**Authors:** Petter Holland, William M. Hagopian, A. Hope Jahren, Tor Erik Rusten

**Affiliations:** 1 Centre for Cancer Cell Reprogramming, Faculty of Medicine, Institute of Clinical Medicine, University of Oslo, Montebello, N-0379 Oslo, Norway; 2 Department of Molecular Cell Biology, Institute for Cancer Research, Oslo University Hospital, Montebello, N-0379 Oslo, Norway; 3 Centre for Earth Evolution and Dynamics, University of Oslo, Blindern, N-0315 Oslo, Norway

**Keywords:** Metabolite, Flux, IRMS, Carbon, Transfer, Food, Host, Tumor, CATSIR

## Abstract

**Background:**

Radioactive or stable isotopic labeling of metabolites is a strategy that is routinely used to map the cellular fate of a selected labeled metabolite after it is added to cell culture or to the circulation of an animal. However, a labeled metabolite can be enzymatically changed in cellular metabolism, complicating the use of this experimental strategy to understand how a labeled metabolite moves between organs. These methods are also technically demanding, expensive and potentially toxic. To allow quantification of the bulk movement of metabolites between organs, we have developed a novel application of stable isotope ratio mass spectrometry (IRMS).

**Results:**

We exploit natural differences in ^13^C/^12^C ratios of plant nutrients for a low-cost and non-toxic carbon labeling, allowing a measurement of bulk carbon transfer between organs in vivo. IRMS measurements were found to be sufficiently sensitive to measure organs from individual *Drosophila melanogaster* larvae, giving robust measurements down to 2.5 μg per sample. We apply the method to determine if carbon incorporated into a growing solid tumor is ultimately derived from food or host tissues.

**Conclusion:**

Measuring tumor growth in a *D. melanogaster* larvae tumor model reveals that these tumors derive a majority of carbon from host sources. We believe the low cost and non-toxic nature of this methodology gives it broad applicability to study carbon flows between organs also in other animals and for a range of other biological questions.

**Supplementary Information:**

The online version contains supplementary material available at 10.1186/s12915-021-01012-5.

## Background

An expanding solid tumor has an extraordinary requirement for nutrients to supply anabolic biosynthetic pathways, mostly in the form of carbohydrates and amino acids. The degree to which solid tumor progression depend on nutrients from host feeding is variable for different types of tumors and the metabolic context of the containing organ [1]. Another potential source of nutrients for the tumor is the host itself, obtained by phagocytosis of neighboring cells (entosis), macropinocytosis [2], or driving release of nutrients from other nearby or distant cells [3]. We set out to determine if carbon incorporated by an expanding tumor is ultimately sourced from ingested food or existing host tissues using a well-established *Drosophila melanogaster* malignant tumor model driven by clonal expression of oncogenic Ras^V12^ and loss of the tumor suppressor scribble [3, 4].

We sought a method that is agnostic to the identity of incorporated carbon metabolites and the modification of metabolites in metabolic pathways of different organs in vivo. Existing methods like radioactive ^14^C-tracing or ^13^C detection through mass spectrometry could allow us to follow a selected metabolite by adding it to an experimental system and then looking for the label in the tumor. This methodology has been essential to establish the fundamentals of tumor metabolism, by infusing labeled metabolites into cell culture media or the blood of an animal [5]. The metabolite that is best known for having increased tumor uptake relative to surrounding tissue is glucose, linked to increased anaerobic glycolysis, exploited in positron emission transmission (PET) imaging and causing secretion of lactate. Labeled lactate has also recently been described to be integrated by tumor metabolism in vivo [6], possibly secreted by poorly oxygenated regions of a tumor and oxidized in well-oxygenated regions [7, 8]. Existing applications of these methods allow a measurement of tumor uptake of a labeled metabolite, but there is no demonstrated application of this methodology to study the transfer of metabolites from other host organs to a tumor in a living animal. Moreover, labeled metabolites are expensive and only allow relative measurements between samples for one metabolite per experiment, not absolute measurements of the mass transfer of carbon.

To allow this type of measurement, we developed an experimental methodology that we have named CArbon Transfer measured by Stable Isotope Ratios (CATSIR), which exploits differences in the abundance of ^13^C/^12^C of biomolecules in edible plants to allow low-cost and non-toxic tracking of the carbon in metabolites. The ^13^C/^12^C is expressed in the delta notation (δ^13^C) in units of per mil (‰) and reported relative to the international standard Vienna Pee Dee Belemnite (VPDB). Plants can be categorized into two main groups with distinct pathways of photosynthesis that results in different δ^13^C values of plant material. The two groups are called C3-type with δ^13^C ≅ − 27‰ (i.e., potato and beets) and C4-type with δ^13^C ≅ − 12‰ (e.g., corn and sugar cane) [9].

The absolute differences in ^13^C between C3 and C4 plants are small, but can be accurately quantified by stable isotope ratio mass spectrometry (IRMS), routinely performed by biologists and biogeochemists to study these plants and how they interact with their environment. IRMS has also been used experimentally in biomedical research to measure the turnover of metabolites within an organ in vivo, for example by feeding mice lipids derived from different plant sources [10, 11]. We here demonstrate a novel application of IRMS that allows a measurement of the transfer of bulk carbon metabolites between organs in vivo.

## Results

### C3- and C4-plant based fly food for stable isotopic labeling of *D. melanogaster*

**Fig. 1:**
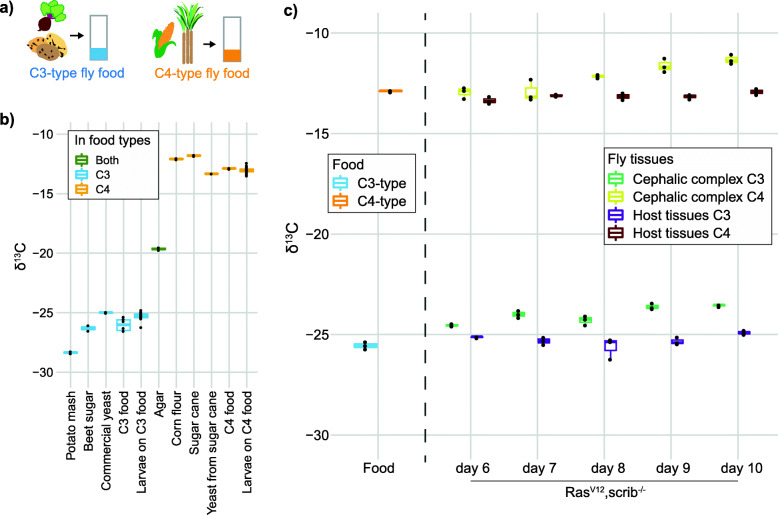
C3- and C4-plant based fly food for stable isotopic labeling of *D. melanogaster*. Food composition (**a**) and δ^13^C of the food components and flies developed on the indicated food types (**b**). Individual food components in **b** are measured in duplicate or singlicate (for C4 yeast). The C3 and C4 composite food types are measured with six replicates and the larvae growing on the two food types are measured with 23 biological replicates. **c** ^13^C/^12^C measurements of the cephalic complex (eye disc and brain) from fly larvae with genetically programmed (Ras^V12^,scrib^−/−^) tumors in the eye disc. Quantified as δ^13^C units per mil (‰), relative to the international ^13^C/^12^C standard Vienna Peedee Belemnite (VPDB). “Host tissues” is a measurement of the remaining organs of the larvae after removing the cephalic complex. Measurements of the food used in these experiments are also included. The larvae organ measurements in **c** are from three biological replicates for each indicated time of larvae development. Each datapoint represents the SIRMS measurement of a single animal. Box plot are used for visualizing the data with default settings for geom_boxplot in R; the median as a line inside boxes extending from the 25th percentile to the 75th percentile and whiskers extend maximally to 1.5x of the inter-quartile range

*D. melanogaster* larvae require a food source containing carbohydrates, amino acids, and lipids for optimal growth. This is achieved by mixing sources of sugar, complex carbohydrates, and yeast with agar to create a pellet of food where eggs are laid and the larvae develop. We determined the commercial baker’s yeast that we use in our standard fly food as being similar to other C3-type nutrients and used this as a C3-type yeast, mixed with potato mash and beet sucrose to create food with a C3 signature (Fig. 1a). To generate C4 yeast, we expanded commercial yeast on sugar cane sucrose as the carbon source and mixed the C4 yeast with sugar cane sucrose and corn flour to create food with a C4 signature. By having flies lay eggs on this food and allowing the larvae to develop, we obtained fully C3- or C4-labeled larvae (Fig. 1b). The lower limit of carbon required to obtain reliable δ^13^C measurements was found to be around 2.5 micrograms of carbon per sample, allowing us to reliably measure organs from individual animals. The tumor measurements were performed by extracting the cephalic complex containing the brain as well as the eye discs where the tumor is growing in our genetic tumor model. It is necessary to take the whole cephalic complex because as the tumor expands, it outgrows the eye discs and invades the brain, making dissection of only the tumor or eye discs impossible at later stages of tumor development.

In our early trials measuring tumors in animals growing only on either C3 or C4 food, we found that as a tumor grows on one type of food, the measured δ^13^C of the cephalic complex gradually becomes less negative, while the other host tissues of the same larvae do not change (Fig. 1c). We found the rate and relative amount of ^13^C enrichment by the tumors to be similar for larvae growing on either the C3 or C4 food. A recent study that measured δ^13^C of human breast cancer biopsies also found an enrichment of ^13^C in human tumor biopsies relative to neighboring control tissue from the same patient [12]. They observed an enrichment resulting in the δ^13^C values to be increased by ~ 3‰ compared to adjacent tissue from the same patient, an effect size in the same range as what we see in our fly model (Fig. 1c). The enrichment of ^13^C by transformed cells was also seen in cell culture of commonly used cell lines of both human and mouse origin [12] and thus appears to be an inherent feature of transformed cells. The metabolic explanation for the observed tumor ^13^C enrichment was suggested to be due to changes in lipid and/or anaplerotic metabolism [12].

### CATSIR—a method to differentiate if carbon incorporated into a growing tumor is derived from ingested food or from host tissues

**Fig. 2:**
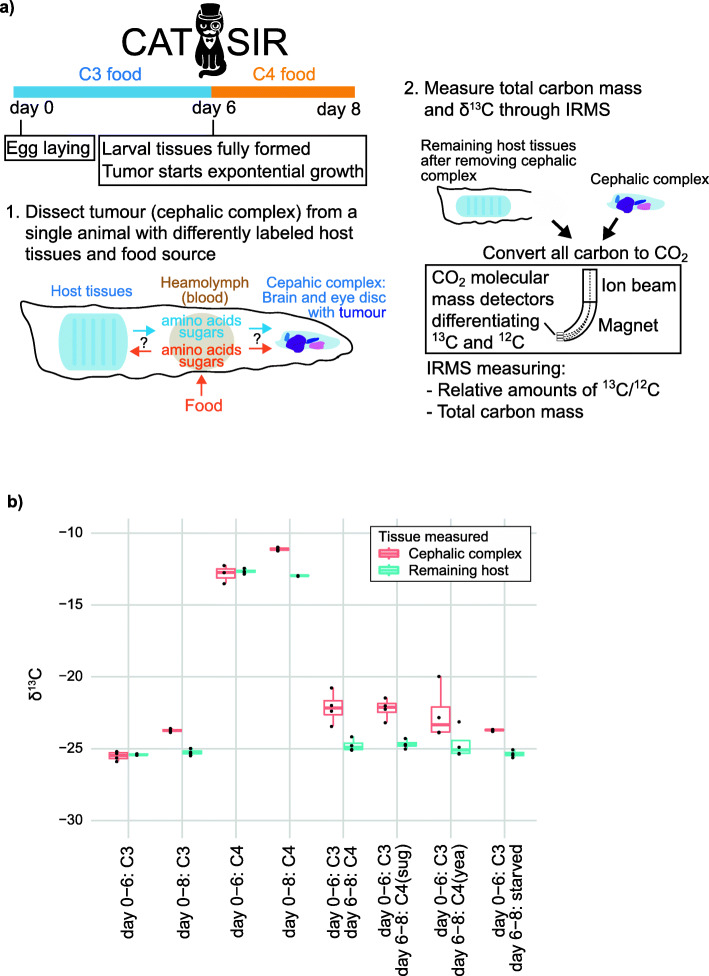
CATSIR, a method to differentiate if carbon incorporated into a growing tumor, is derived from ingested food or from host tissues. **a** The protocol starts with genetic crosses of flies that will generate offspring with tumors in the eye-antennal disc. Larvae develop on C3-type food from egg laying until day 6, when the larvae are fully developed. At day 6, the exponential growth of the tumor starts and the larvae is then moved to C4-type food, obtaining a situation where the host tissues have C3-labeled carbon and ingested food nutrients have C4-labeled carbon. At day 8, the carbon composition as well as total carbon mass of the tumor is measured, allowing a determination of where the growing tumor is obtaining carbon to expand its biomass. **b** δ13C measurements of indicated tissue from Ras^V12^,scrib^−/−^ larvae. In addition to the standard C4 food, larvae were moved to other food variants – one with only sugars (C4 sug), one with only yeast (C4 yea) and one without any nutrients (starved). See also Additional file 1: Figure S1 for an illustration using simulated data of how the δ13C measurements can be used to determine where a growing tumor is obtaining carbon

To differentiate if carbon incorporated into an expanding tumor biomass is ultimately derived from ingested food or the host, we need an experimental situation where the carbon in the food is labeled differently than the carbon in host tissues. We can achieve this in the Ras^V12^,scrib^−/−^ *D. melanogaster* tumor model if the food source is changed at day 6 (from C3-type to C4-type), when the host tissues are fully developed, but the tumor is very small (Fig. 2a). Pupation (the transformation from larvae to adult fly) normally starts around day 6 for these animals, but the growing tumor delays this process, giving an experimental window of several days starting from day 6 when the food and host tissues will have a different carbon composition, the host tissues are isotopically stable (Fig. 1c), and the tumor is growing exponentially.

**Fig. 3:**
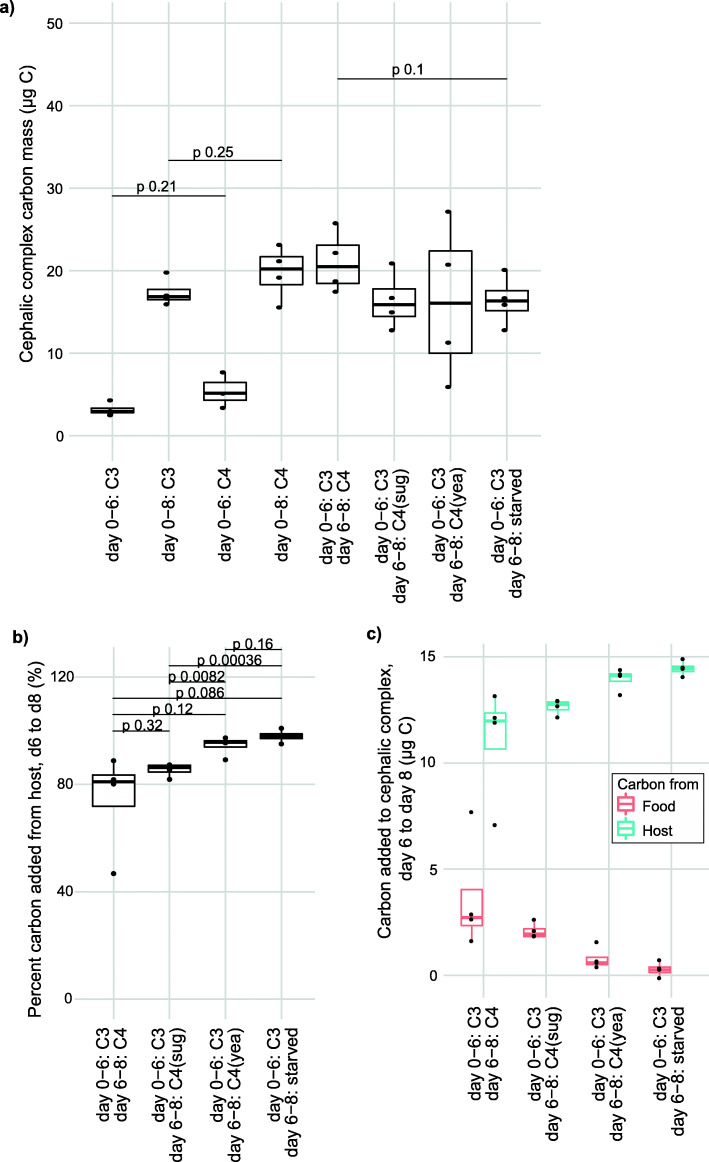
CATSIR applied to measure the sources of carbon used for tumor growth in *D. melanogaster* larvae. **a** Cephalic complex total carbon mass measurements of the same larvae as in Fig. 2b. **b** The calculated amounts of carbon from the food or host tissues being incorporated into the tumor between day 6 and 8, calculated from the SIRMS data shown in Figs. 2b and 3a. **c** Percentage of carbon added between day 6 and day 8 derived from host sources. All measurements in Fig. 3 are from four independent biological replicates with the exception of three replicates for the C4 day 6 set where one replicate was lost because there was not enough carbon in it to obtain a reliable measurement. All indicated statistical test *p* values are from an unpaired two-sided t-test between the indicated groups. Box-plots are used for visualizing the data with default settings for geom_boxplot in R; the median as a line inside boxes extending from the 25th percentile to the 75th percentile and whiskers extending maximally to 1.5x of the inter-quartile range

The observed tumor ^13^C enrichment (Fig. 1c) is a type of isotopic fractionation which means that δ^13^C measurements of the food itself cannot be used to directly calculate how much carbon from ingested food is being incorporated into the growing tumor. Instead, we rely on measuring tumors from animals developing on either of the two food types at multiple stages of development, creating baseline measurements that allow calculations of where a tumor is sourcing its carbon (Figs. 2b, 3a). The baseline measurements are used as references to determine the carbon sources without having to correct explicitly for isotopic fractionation in the calculations. Importantly, we found that the cephalic complex mass is not significantly different between larvae growing only on C3 or C4 food at neither day 6 or 8 (Fig. 3a, *p* = 0.21 at day 6, *p* = 0.25 at day 8), meaning the two food types are similarly able to support tumor growth.

### Quantifying tumor incorporation of carbon from host and food sources in *D. melanogaster* larvae

We illustrate how the δ^13^C measurements can be used to determine the source of carbon used for tissue growth with simulated data in Additional file 1: Figure S1. Incorporating the baseline measurements, we solve two separate equations to calculate where the carbon incorporated into the tumor from day 6 to day 8 is coming from, one determining the isotopic composition of the cephalic complex if the growth was incorporating only food-derived carbon and the other equation gives the isotopic composition of the cephalic complex if the tumor was incorporating only host-derived carbon. For the larvae that were moved from C3 to C4 food on day 6, the experimentally measured isotopic composition of the cephalic complex at day 8 (Fig. 2b) is compared to these two theoretical values to create a factor of the relative contribution from the food and host (Fig. 3b). The measured carbon mass added between day 6 and day 8 (Fig. 3a) is multiplied by this factor to calculate the mass of carbon added to the tumor from the two sources (Fig. 3c). Using this approach, we found that Ras^V12^,scrib^−/−^-driven tumor growth between day 6 and day 8, tumor growth that causes a 4-fold increase in the cephalic complex carbon mass (Fig. 3a), sources a majority of the carbon it uses for biomass expansion from host sources as well as a smaller amount directly from the food (Fig. 3b, c). As a control, we also performed the reverse experiment where larvae first developed on C4 food and were moved to C3 food for 2 days and we found the measured amount of carbon coming from host sources relative to food sources is the same if the larvae go from C4-to-C3 vs C3-to-C4 (Additional file 1: Figure S2 a-c).

This methodology can further be adapted to allow measurements of how the different carbon sources in the food change the amount of food-derived carbon that is incorporated in tumor growth. Moving the larvae at day 6 to food with C4 sugar (no yeast) demonstrated a relative contribution to growth from the food and host carbon that is similar to what is seen for a complete C4 food, while having only C4 yeast (no sugars) trends towards having less carbon incorporation into the tumor from the food (Fig. 3b). Having no nutrients (only agar, starved) in the food demonstrated no incorporation of carbon from the food (Fig. 3c). We were surprised to see that the tumor growth itself was not significantly reduced when various nutritional components are removed from the food, even when there are no nutrients (Fig. 3a, *p* = 0.1). This observation, seen together with the large amount of incorporation of host-derived carbon also when food is present (Fig. 3b, c), is a key insight from these experiments that point to a close interaction between tumor and host metabolism in vivo*.*

## Discussion

The CATSIR methodology allows a new type of measurement of bulk carbon transfer in vivo and expands the methodological arsenal of researchers studying systemic metabolism. Rather than competing with or replacing existing methods, we think CATSIR has strong synergy with other types of metabolite flux measurements that rely on labeling selected metabolites by radioactivity or stable isotopes. CATSIR also uniquely allows a direct measurement of carbon mass, information that is not typically available through detection of specifically labeled metabolites, where the read-out is a relative measurement of the label between samples. We imagine an experimental strategy that starts with many low-cost CATSIR experiments and then following up on selected candidates with detailed studies using isotopic or radioactive tracers would be a way to maximize the biological insights about metabolite flows through systemic metabolism.

We observe an enrichment of ^13^C by tumors compared to other measured tissues of the same animal, supporting a recent finding from studies of human tumor biopsies by Tea et al. [12] They expanded on this observation with a series of additional experiments and suggest that the 13C enrichment is due to changes in lipid content and/or anaplerotic incorporation of ^13^C-enriched carbon into tumor biomass. Seeing the same phenomena in our genetically induced *D. melanogaster* tumors is strong support of this being a universal feature of transformed cells and explaining this should be a fruitful avenue for further research, possibly elucidating unknown fundamental features of tumor metabolism.

When changing the amounts of different categories of nutrients in the food, we found that removing the yeast (amino acid and lipid source, samples labeled C4(sug)) had a minimal effect on the amount of carbon the tumor is integrating from food sources. In contrast, there appears to be some effect from removing the sugar from the food (samples labeled C4(yea)), with these samples trending towards less integration of carbon from the food. It is apparent that the tumor is able to obtain all the nutrients it needs from host sources because it grows well even without any nutrients in the food. The tumor is well known to have an exceptional need for circulating sugars and our impression from these experiments is that the tumor is highly opportunistic, using sugars from ingested food when they are available, but able to source sugars or equivalent metabolites from host sources if they are not available in the food. Further experiments are needed to be able to conclude on the nutritional interactions between a tumor and different food nutrients and the CATSIR methodology should be useful for this.

The main limitation on the use cases of CATSIR is that the method requires organ growth in the experimental interval when the food is changed. However, the general principle of using C3- and C4-based food to label tissues in vivo and IRMS measurements allow other types of studies that are not limited to measuring organ growth. One possibility is to mix C3 and C4 food components together and measure the incorporation of carbon from the C3 and C4 sources into different organs, allowing insights about the preference of different organs for different categories of metabolites. Another possible application is to use the C3 and C4 labeling to give a measurement of the anabolic results of feeding within an organism —the incorporation of the food-derived nutrients in biomass.

## Conclusion

Here, we successfully employ CATSIR to measure the mass of carbon incorporated into a growing tumor from host and food sources in a *D. melanogaster* tumor model. The demonstrated strategy should be adaptable to in vivo studies of any animal because of the sensitivity, simplicity, low cost, and non-toxic nature of the carbon labeling. Through technical optimizations, we achieved reliable measurements down to 2.5 μg of total carbon per sample, making the methodology applicable for smaller samples like biopsies that are relatively easily obtained. More generally, the use of C3- and C4-based food to label tissues and subsequent IRMS measurements is low-cost unexplored experimental strategy that should allow new types of measurements for a range of biological questions.

## Methods

### Fly food

C3 food was prepared with 32.7 g/L potato mash, 60 g/L beet-derived sucrose, and 27.3 g/L commercial dry yeast (Lesaffre, Saf-instant. *δ*^13^*C* measured to be similar to C3-type plants). C4 food was prepared with 32.7 g/L corn flour, 62 g/L cane-derived sucrose, and 26.3 g/L commercial yeast that was expanded on sucrose from sugar cane. The amount of sugar cane and yeast added to the C4 food was slightly adjusted compared to the C3 food to account for the higher protein content and lower carbohydrate content of the corn flour compared to the potato mash, giving a similar final fat, protein, and carbohydrate content of the two food variants. Both foods were also added 4.55 ml/L propionic acid (Sigma, P5561), 2 g/L nipagin (Sigma, H5501) and 7.3 g/L agar (AS Pals, 77,000).

### Fly genetics

Larvae with genetically programmed tumors in the eye disc were generated by crossing y,w,ey-flp; Act>y+>Gal4, UAS-GFP/CyO; Frt82B, tub-Gal80 females with y,w;UAS-Ras^V12^/CyO; Frt82B,scrib/TM6B males.

### Sample preparation

When moving larvae from C3 to C4 food, holes were poked in surface of the new food to give easy immediate access to the new food. Before dissection, larvae were washed 3 times in water and the cephalic complex (eye disc and brain) was dissected in a drop of ultrapure water. The cephalic complex from single larvae was added to a tin capsule (Elemental microanalysis, D1006), and the remaining tissues of the larvae after removing the cephalic complex was added to a separate tin capsule. Samples were then left to dry in a desiccator.

### Stable isotope measurement

The carbon stable isotope value of each sample was determined using a Delta V Advantage Isotope Ratio Mass Spectrometer (Thermo Fisher, Bremen, Germany) configured with a Thermo Fisher EA Isolink Elemental Analyzer at the University of Oslo, Norway. Samples were loaded into a zero blank autosampler (Costech Analytical, Valencia, USA) and quantitatively combusted to CO_2_ via Dumas combustion in the elemental analyzer. The CO_2_ flowed to the mass spectrometer within a stream of helium where the ^13^C/^12^C were determined. Carbon stable isotope values were expressed in the delta notation (δ^13^C) in units of per mil (‰).

We tuned the instrument source to obtain high sensitivity while still maintaining acceptable linearity. This resulted in the following source parameters: emission current = 1.50 mA, trap current = 40.0 V, electron Energy = 124.000 eV, and extraction = 75.19%. The oxygen injection on the EA was set to 6 mL O_2_, enough to fully oxidize the tin capsule (average mass = 8.5 mg) and the carbon in the samples. The helium carrier gas flow rate was set to 140 mL/minute. For samples containing < 40 μg C, sample helium dilution was set to 0 to maximize the amount of CO_2_ flowing to the spectrometer. This resulted in m/z 44 peak intensities ranging from 300 to 6400 mV (with corresponding peak areas of 6 to 125 Vsec) for sample amounts between 2 and 40 μg C. Peak to baseline separation was excellent down to the lower limits measured, as shown in Additional file 1: Figure S3 a. Multiple replicates of two internal lab reference materials (“JRICE”, a white rice obtained from a supermarket and homogenized with a ball mill, *δ*^13^C = − 27.43‰,, and “JGLUT”, L-glutamic acid obtained from Fisher Scientific, *δ*^13^C = − 13.43‰) were incorporated into each analytical batch run and used to normalize the data to the Vienna Pee Dee Belemnite (VPDB) scale using a standard regression method [13]. Additionally, quality control samples (“JALA”, L-Alanine from Fisher Scientific, *δ*^13^C = − 20.62‰) were incorporated into every batch run and analyzed as unknowns. All three materials (JRICE, JGLUT, JALA) were calibrated within the CLIPT laboratory using the IAEA internationally recognized standards, LSVEC, and NBS- 19, which define the VPDB scale [14]. To verify that our calibrations were accurate, we analyzed IAEA-601 benzoic acid (consensus *δ*^13^C = − 28.81‰) as an unknown and obtained *δ*^13^C = − 28.83 ± 0.04‰ (1휎., *n* = 6).

For samples containing < 40 μg C, carbon blank contributions from the sample tin capsules becomes significant, which can bias the sample *δ*^13^C towards the value of the carbon blank [15–17]. Additionally, linearity effects inherent to the instrument can skew *δ*^13^C values as sample sizes decrease [18, 19]. We assessed and corrected for both of these effects as follows:

#### Blank correction

Prior to the project, we assessed several types of tin capsules, ranging in size and manufacturer, to find the one with the lowest and most consistent carbon blank. We settled on 4 × 3.2 mm tin capsules (catalog # D1000, Elemental Microanalysis, UK) with 0.31 ± 0.03 μg C blank, and *δ*^13^C = − 27.69 ± 0.22‰ (raw *δ*^13^C value used for blank correction). Empty tin capsules were incorporated throughout the sample analyses and peak areas and *δ*^13^C of the carbon in the tin capsules were measured directly as recommended by Polissar [16]. The blank contribution to each sample was removed using a simple mixing model [16]:

$$ {\delta}^{13}{C}_S=\frac{\ {A}_M{\delta}^{13}{C}_M-{A}_B{\delta}^{13}{C}_B\ }{A_M-{A}_B} $$

where *δ* ^13^C_S_ is the blank corrected sample value, A_M_ is the measured total area of the sample, *δ*^13^C_M_ the measured *δ* ^13^C of the sample, A_B_ the average measured area of empty capsules, and *δ*^13^C_M_ the average measured *δ*^13^C value of the empty capsules.

#### Linearity correction

To assess and correct for linearity effects, either two or three size series composed of different glucose materials with differing isotopic compositions were incorporated into each analytical run: GLUC1 (− 13.43‰), GLUC2 (− 19.93‰), and GLUC3 (− 26.38‰). GLUC 1 was ACS grade glucose (Thermo Scientific, USA) and GLUC2 and GLUC3 were created by mixing GLUC 1 with D-Glucose-^12^C_6_ (99.5% pure, Sigma-Aldrich, USA).

The size series were created by generating an aqueous solution of glucose containing 10 μg glucose to 1 μL H_2_O and then performing a serial dilution to obtain solutions containing between 0.5 and 100 μg glucose to 1 μL H_2_O. Ten microliter aliquots of each solution were then added to empty tin capsules, resulting in a series of capsules containing between 2 and 40 μg of carbon once the water evaporated. GLUC1, GLUC2, and GLUC3 were calibrated within our laboratory using LSVEC and NBS- 19. For each batch run, we created a linearity correction equation by plotting total mass spectrometer peak area versus blank corrected *δ* ^13^C for each glucose size series, and then using the average of the regression equations to remove the linearity effect. An example blank and linearity corrected GLUC1 size series is shown in Additional file 1: Figure S3 b.

After removing the blank and linearity effects, all data was normalized to the VPDB scale using JGLUT and JRICE reference materials that were included in each analytical run. Normalized values of JALA, GLUC1, GLUC2, and GLUC3 analyzed as unknowns are shown in Table 1.

**Table 1:** Corrected and calibrated values of reference materials analyzed as unknowns for samples containing 2 to 40 μg C

Material	*δ*^13^C calibrated^a^	*δ*^13^C measured (all reps)	*δ*^13^C measured (2–3 μg C reps only)^b^
JALA	− 20.62 ± 0.03 (25)	− 20.59 ± 0.06 (91)	N/A
GLUC1	− 12.45 ± 0.04 (10)	− 12.44 ± 0.13 (84)	− 12.50 ± 0.17 (16)
GLUC2	− 19.93 ± 0.03 (10)	− 19.91 ± 0.07 (84)	− 19.91 ± 0.10 (16)
GLUC3	− 26.38 ± 0.04 (10)	− 26.37 ± 0.06 (35)	− 26.33 ± 0.07 (12)

Mean values ±1 standard deviation. Number of measurements reported in parentheses. ^a^Calibrated against IAEA standards LSVEC and NBS-19. ^b^Included to illustrate accuracy and precision at the lowest carbon amounts measured

We calculated micrograms of carbon in each sample using the total mass spectrometer peak areas. For samples containing < 40 μg C, a standard curve was generated from the GLUC1 size series which enabled us to determine the amount of carbon in each combusted capsule down to 2.0 μg ± 5%. For samples containing > 40 μg C, we used a standard curve generated from JALA peak areas that spanned the range of the unknowns.

### Carbon transfer calculations

To calculate the relative contribution of carbon from the food and host tissues to tumor growth between days 6 and 8, the carbon mass and δ^13^C needs to be measured for larvae growing only on C3 food at day 6 (${\delta}^{13}{C}_{C{3}_{d6}}$) and day 8 (${\delta}^{13}{C}_{C{3}_{d8}}$) as well as larvae growing only on C4 food at day 8 (${\delta}^{13}{C}_{C{4}_{d8}}$). When an experimental larva is moved from C3 to C4 food at day 6 and then measured at day 8, the amount of tumor growth between day 6 and day 8 is calculated by subtracting the measured cephalic complex carbon mass at day 8 (${\mu gC}_{C3-C{4}_{d8}}$) from the mean cephalic complex mass at day 6 (${\mu gC}_{C{3}_{d6}}$) giving *μgC*_growth_. Two theoretical δ^13^C values are then calculated to determine what the isotopic composition would be at day 8 if all the carbon for growth between day 6 and day 8 was coming from the food (*δ*^13^*C*_allFood_) or if all carbon was coming from the host (*δ*^13^*C*_allHost_):

$$ {\delta}^{13}{C}_{\mathrm{allFood}}=\frac{\ {\delta}^{13}{C}_{C{3}_{d6}}\ast {\mu gC}_{C{3}_{d6}}+{\delta}^{13}{C}_{C{4}_{d8}}\ast {\mu gC}_{\mathrm{growth}}\ }{{\mu gC}_{C{3}_{d6}}+{\mu gC}_{\mathrm{growth}}} $$

$$ {\delta}^{13}{C}_{\mathrm{allHost}}=\frac{\ {\delta}^{13}{C}_{C{3}_{d6}}\ast {\mu gC}_{C{3}_{d6}}+{\delta}^{13}{C}_{C{3}_{d8}}\ast {\mu gC}_{\mathrm{growth}}}{{\mu gC}_{C{3}_{d6}}+{\upmu \mathrm{gC}}_{\mathrm{growth}}} $$

The measured δ^13^C of the cephalic complex at day 8 (${\delta}^{13}{C}_{C3-C{4}_{d8}}$) is then compared to these two theoretical values:

$$ \mathrm{fractionFood}=\left({\delta}^{13}{C}_{\boldsymbol{allHost}}-{\delta}^{13}{C}_{C3-C{4}_{\mathrm{d}8}}\right)/\left({\delta}^{13}{C}_{\mathrm{allHost}}-{\delta}^{13}{C}_{\mathrm{allFood}}\right) $$

$$ \mathrm{fractionHost}=1-\mathrm{fractionFood} $$

Finally, these fractions are multiplied by the measured carbon mass added to each sample between day 6 and day 8 to derive the total amount of carbon added from the food or host:

$$ {\mu gC}_{C3-C{4}_{d8}}\mathrm{food}={\mu gC}_{\mathrm{growth}}\ast \mathrm{fractionFood} $$

$$ {\mu gC}_{C3-C{4}_{d8}}\mathrm{host}={\mu gC}_{\mathrm{growth}}\ast \mathrm{fractionHost} $$

## Supplementary Information

**Additional file 1: Figure S1.** Simulated data to illustrate how the δ^13^C measurements are used to differentiate the sources of carbon. **Figure S2.** CATSIR performed with the reversed order of food changes as compared to the experiments shown in Figs. 2 and 3. **Figure S3.** SIRMS instrument validation and optimization.

**Additional file 2.** “IRMS raw data.xlsx” – IRMS measurements raw data. “IRMS calculation R scripts. R” – R scripts for the required calculations and creating the figures in the manuscript.

## Data Availability

Raw data from IRMS measurements as well as R scripts that contain all required calculations and scripts to directly reproduce the figures in the manuscript from the raw data are supplied in “Additional file 2.zip”. The raw IRMS spectra will be made available via Isobank, a database for isotope data that is currently under development (https://isobank-qa.tacc.utexas.edu/).
