# Supplementary material for: Natural abundance isotope ratios to differentiate sources of carbon used during tumor growth in vivo
Source: BMC Biol. 2021 May 10;19:85. doi: 10.1186/s12915-021-01012-5 (PMC8108461; doi:10.1186/s12915-021-01012-5)
Supplement: Supplementary file 1 — Additional file 1: Figure S1. Simulated data to illustrate how the δ13C measurements are used to differentiate the sources of carbon. Figure S2. CATSIR performed with the reversed order of food changes as compared to the experiments shown in Figs. 2 and 3. Figure S3. SIRMS instrument validation and optimization. [file 12915_2021_1012_MOESM1_ESM.docx]

**Supplementary Figure 1**: Simulated data to illustrate how the δ^13^C measurements are used to differentiate the sources of carbon. ^13^C/^12^C measurements are quantified as δ^13^C units per mil (‰), relative to the international ^13^C/^12^C standard Vienna Peedee Belemnite (VPDB). The tumor grows from day 6 to day 8 with host tissues labelled as C3 and ingested food labelled as C4. As the tumor δ^13^C of a larvae that was moved from C3 to C4 food on day 6 is measured at day 8, the distance between the δ^13^C of a day 8 tumor growing only on C3 food and a day 8 tumor growing in only C4 food determines the ratio of carbon derived from the two sources. The ratio can further be integrated with a measurement of the amount of carbon that has been added to the tumor between day 6 and day 8 to determine the absolute amount of carbon added from host and food sources.

**Supplementary Figure 2**: CATSIR performed with the reversed order of food changes as compared to the experiments shown in Figure 2 and Figure 3. a) Larvae developed from day 0 to day 5.5 on C4 food and then moved to C3 food for 2 days before tumor measurement at day 7.5. b) The carbon mass added between day 5.5 and 7.5 from host and food sources. c) The percentage of carbon added to the tumor from host sources for the C4-to-C3 experiment compared to the C3-to-C4 experiment (shown in Figure 3). The carbon added for the C4-to-C3 experiment between day 5.5 and day 7.5 of larval development is less than the C3-to-C4 experiment between day 6 and day 8 (compare Supplementary Figure 2 b to Figure 3 c) because of the different time-points and exponential tumor growth. Each datapoint represents the IRMS measurement of a single animal. Box-plot are used for visualizing the data with default settings for geom_boxplot in R; the median as a line inside boxes extending from the 25^th^ percentile to the 75^th^ percentile and whiskers extend maximally to 1.5x of the inter-quartile range. The indicated statistical test p-value in a) is from an unpaired two-sided t-test.

**Supplementary Figure 3**: IRMS instrument validation and optimization. a) Mass spectrometer m/z 44 trace of blank contribution from an empty tin capsule, and a GLUC1 sample containing 2 µg of carbon. b) Glucose size series comparing raw *δ*^13^C measured values to blank and linearity corrected values. The blank and linearity corrected values are subsequently normalized to the international VPDB scale using internal reference materials JGLUT and JRICE.
